# Oral health knowledge, attitude, and practice of pregnant women in Deccan, South India: a cross-sectional prenatal survey

**DOI:** 10.25122/jml-2019-0095

**Published:** 2022-03

**Authors:** Mukhatar Ahmed Javali, Shahabe Abullais Saquib, Mohasin Abdul Khader, Imran Khalid, Abdulrahman Yahya AlShahrani, Masroor Ahmed Kanji, Elyas Asiri

**Affiliations:** 1. Department of Periodontics and Community Dental Sciences, College of Dentistry, King Khalid University, Abha, Saudi Arabia; 2. Department of Oral and Maxillofacial Surgery, College of Dentistry, King Khalid University, Abha, Saudi Arabia; 3. Department of Dentistry, Albashaier Hospital, Ministry of Health, Bisha, Saudi Arabia; 4. Department of Prosthodontics, College of Applied Medical Sciences, King Khalid University, Abha, Saudi Arabia; 5. Department of Dentistry, Ministry of Health Clinics, Abha, Saudi Arabia

**Keywords:** attitude, oral hygiene, practice, pregnancy, knowledge

## Abstract

Pregnancy is a unique condition for women, associated with physiological and emotional changes in the body. Various research showed an association between periodontal disease and adverse pregnancy outcomes. Importance to hygiene maintenance should be given during pregnancy and improve the wellbeing of the mother and child. This study assessed oral health knowledge, attitude, and practices among pregnant women in Hyderabad. The study design was cross-sectional and included 445 women who responded and completed the survey. Subjects were selected using a random sampling technique in gynecology clinics. The questionnaire form consisted of four sections: demographic data, knowledge, attitude, and oral hygiene practice. Out of the 482 pregnant females invited to participate in the study, 445 women completed the survey, giving a response rate of 92%. The majority of women showed good knowledge and attitude regarding oral hygiene and its relation to pregnancy. However, the participants showed poor compliance with the recommended protocol. There are certain myths and barriers to dental treatment that need to be considered in the prenatal education of women. If explained by the gynecologist, the importance of oral health and its correlation with systemic health will play a crucial role in improving oral hygiene practice and regular dental visits.

## INTRODUCTION

Recently, the dental community has stressed the two-way relationship between oral health and general health. Nowadays, the priority is to improve oral health and minimize the negative effect of oral disease on general health [1, 2]. In females, oral health is influenced by physiological events such as pregnancy, menopause, and non-physiological conditions such as contraceptive pills and hormonal replacement therapy (HRT) [3]. Pregnancy is a special condition for a woman associated with physiological and emotional changes in the body [4]. Oral hygiene during pregnancy has been acknowledged globally as a significant health issue. Studies reported various oral pathologies amongst pregnant women [5]. Gingivitis is one of the most common periodontal diseases characterized by inflammation of the gingiva under the influence of bacterial plaque. Dental plaque is a prime etiological agent for dental caries and periodontal disease. During pregnancy, gingiva shows an exaggerated inflammatory response to bacterial plaque, attributed to the increased level of hormones (mainly estrogen and progesterone) [6, 7]. Periodontal disease is preventable and treatable. The key objective of oral health care in pregnancy is to produce a healthy environment with good oral hygiene practices (like tooth-brushing, flossing) and professional oral prophylaxis, including scaling and root planing [6].

Recently, various studies showed a relationship between periodontal disease and adverse pregnancy outcomes such as preterm low birth weight, premature births, and pre-eclampsia [7–9]. A systematic review of 25 studies identified that periodontal infections might be associated with adverse pregnancy outcomes [10]. An Australian study concluded that women with miscarriages compared to women with full-term babies and live-born infants were likely to have four times more periodontal disease [11].

However, many women do not pursue dental supervision, intervention, and treatment during pregnancy [9]. According to a study, around 44.7% of pregnant women in the USA consulted a dental surgeon during their pregnancy, when an oral and dental problem existed [12]. In the Australian population, the dental consultation rate is 30% to 36% among pregnant women [13, 14]. One study indicated that 35–50% of pregnant women visited the dental surgeon during pregnancy [15]. Another study reported that only 35% of women during pregnancy visited the dentist, and 35% had no dental appointments for at least two years prior to their pregnancy [16]. The main types of treatment received during pregnancy were examinations (96%) and routine cleaning (95%). Some barriers between dental visits and pregnant women are pregnancy stressors, unpleasant past dental experience, attitude towards dentist, importance and valuing of oral hygiene, financial restraint, and time constraints [15].

It is recommended for women to have a regular dental visit during pregnancy for a complete oral health check-up and risk evaluation [17]. The existing research found that knowledge and practices of pregnant women in India concerning their oral health were poor [18]. The aim of this study was to assess the knowledge, attitude, and practices of oral health among pregnant women in South India. The results will serve as a guideline for oral health education programs to improve the oral health of pregnant women. We hypothesized that the trimester of pregnancy and level of education influence women's knowledge and choices regarding oral health in pregnancy.

## MATERIAL AND METHODS

This cross-sectional study used a self-explanatory close-ended questionnaire to evaluate pregnant women's oral health knowledge, attitude, and practice from different places across South India. A draft questionnaire was constructed with 15 questions in English and local language. The questionnaire was confined to four sections. The first section contained questions regarding the sociodemographic features of participants such as age, number of pregnancies, and educational status. The second section had eight questions regarding oral health and its importance during pregnancy. The third section consisted of questions regarding attitude towards oral health. The fourth section had six questions regarding oral hygiene practices. The final form was checked for face validity by a linguistic expert. A total of 445 women responded and completed the questionnaire. The questionnaires were distributed to pregnant women in the outpatient department waiting area, with a duration of 20 minutes. Furthermore, the investigator was available during the filling of the questionnaire so that any questions raised by the subjects could be answered. The data were analyzed using descriptive statistics.

## RESULTS

Out of the 482 pregnant women invited to participate in the study, 445 women completed the survey, giving a response rate of 92%. The age ranged from 18 to 38 years, with a mean age of 26.1 years (SD±5.6). The majority of the participants were in the age range of 18–35 years (98.5%). Most participants had 1^st^, 2^nd^ or 3^rd^ pregnancy, which was equal distribution (32%, 28%, and 27.5%). About 65% of the participants had high education status or secondary school. Most participants (95%) were housewives, and a very small number of women were employed. About (60.0%) of the women were in their second trimester of pregnancy (Table 1).

**Table 1.** Demographic data of the participants.

**Age**	
<25	56.0%
25–35	42.5%
>35	1.5%
**Number of Pregnancy**	
1^st^	32.0%
2^nd^	28%
3^rd^	27.5%
4^th^	11.0%
5^th^	1.5%
**Level of Education**	
No schooling	10%
Pre-secondary	25%
Secondary	12%
Higher secondary	10%
Graduation	33%
Post-graduation	10%
**Employment status**	
Working	5%
Housewife	95%
**Trimester of pregnancy**	
1^st^	26%
2^nd^	60%
3^rd^	14%

47% of the pregnant women believed that caries is caused by the sugar contained in food, and about 74% of the pregnant women considered bacteria and tartar the prime cause of bleeding gums. According to the majority (62.5%) of the participants, the appropriate time for dental treatment is before the start of pregnancy. When participants were asked about the effect of X-rays and medication on pregnancy, most of them (92.5% and 97%, respectively) agreed that there was a positive effect. 54% of the total participants considered gum diseases a major cause of bad breath (Table 2). The majority of participants believe that dental health and attendance improve the quality of general health (95.5%) and that pregnant women need dental check-ups (97%) (Table 3). Most participants in the positive attitude group had higher education, were 18 to 25 years old, and were first-time pregnant.

**Table 2.** Distribution of self-reported knowledge of oral health and its importance during pregnancy.

Question no.	Total participants (n=445)	Option	Number of responses (n)	% of responses
**Q1 What are the reasons for tooth decay?**		a. Improper brushing	216	48.5%
		b. Food containing sugar	208	47%
		c. Weak tooth structure	20	4.5%
**Q2 What are the reasons for bleeding gums?**		a. Dental cavities	106	24%
		b. bacteria and tartar	330	74%
		c. Irregular placed teeth	8	2%
		d. Others.	0	0%
**Q3 Appropriate time for dental treatment is……**		a. Before pregnancy	275	62.5%
		b. During pregnancy	25	5.5%
		c. After pregnancy	142	32%
**Q4 Do X-rays have an effect on the mother and baby?**		a. Yes	412	92.5%
		b. No	32	7.5%
**Q5 Do medications have an effect on the mother and baby?**		a. Yes	431	97%
		b. No	13	3%
**Q6 Bad breath is caused by……**		a. Dental caries	136	30.5%
		b. Gum disease	242	54%
		c. Bacteria and tartar	50	11%
		d. Foods	16	3.5%
		e. Tongue coating	00	0%

**Table 3.** Distribution of attitude towards oral health reported by participants.

**Question no.**	**Total participants (n=445)**	**Option**	**Number of responses (n)**	**% of responses**
**Q1 Does good dental health improve the quality of general health?**		a. Yes	423	95.5%
		b. No	21	4.5%
**Q2 Do pregnant women need a dental check?**		a. Yes	432	97%
		b. No	12	3%

Regarding their oral hygiene practices, the majority of women (80.5%) brushed their teeth once daily, and 18.5% cleaned their teeth twice daily; they mainly belonged to the higher education group (78.2%) and housewives (83.9%). About 67% of the participants brushed their teeth only in the morning, and 13% brushed in the morning and before bed. Majority of women from the 3^rd^ and 4^th^ pregnancy group and participants in the third trimester used to brush on other times. Half of the participants (50.5%) brushed their teeth for only one minute and about 37% for less than one minute. These options were selected by employed women with secondary and higher education and who had 3^rd^ or 4^th^ pregnancy (Table 4).

**Table 4.** Distribution of oral hygiene practice reported by participants.

Question no.	Total participants (n=445)	Option	Number of responses (n)	% of responses
**Q1 How often do you brush your teeth?**		1. Not once/day	4	1%
		2. Once/day	358	80.5%
		3. Twice/day	82	18.5%
		4. More than twice/day	00	0%
**Q2 When do you brush your teeth?**		1. Morning	300	67%
		2. Noon (after lunch)	00	0%
		3. Before going to bed and in the morning	56	13%
		4. Other times (specify)	88	20%
**Q3 For how long do you brush your teeth?**		1. Less than one minute	164	337%
		2. One minute	226	50.5%
		3. Two minutes	45	10%
		4. More than two minutes	10	2.5%
**Q4 Type of brush used.**		1. Soft	56	13%
		2. Medium	278	62.5%
		3. Hard	44	10%
		4. Ultra-soft	64	14.5%
**Q5 Type of toothpaste used.**		1. Fluoridated	404	90.5%
		2. Non-fluoridated	00	0%
		3. Others	38	9.5%
**Q6 Visit to the dentist.**		1. Others	00	0%
		2. Once in 3 months	00	0%
		3. Once in one year	80	18%
		4. Every 3–5years	4	1%
		5. Only if there is pain	360	81%

62.5% of the studied population used medium bristle brushes to brush their teeth. About 90.5% of women were using fluoridated toothpaste. Women with lower educational levels knew less about the beneficial effects of fluoride toothpaste. About 81% of the participants chose to visit a dentist only if there was tooth pain. Women from higher education groups and employed chose to visit a dentist once a year, making up only 18% of total participants (Table 4).

## DISCUSSION

Good oral hygiene throughout pregnancy is important, especially given recent studies suggesting an association between deprived oral health and adverse pregnancy outcomes [7–9]. This is even more important in India because of the high mortality rates of pregnant women. The most common oral disease in pregnancy (*i.e.* periodontal disease) is avoidable by establishing simple oral hygiene measures. However, such positive conduct is influenced by individuals' knowledge and attitudes towards oral health. Thus, this study was planned to provide an insight into oral health knowledge, attitudes, and practices of pregnant women in India.

Knowledge about oral hygiene maintenance, such as brushing two times a day, using fluoridated toothpaste, brushing for at least 2 to 3 minutes, and routine dental visits at least two times a year, was not satisfactory. Knowledge about oral health and reinforcement of oral hygiene techniques might be necessary.

In the present study, the mean age of the participants was 26.1 years (SD±5.6), and only 32% were first-time pregnant, and the rest were multiparous. Similar results were observed in other studies of Indian origin [18]. Studies from European and African countries reported that the mean age of the participants was 30 years, and 57% were having their first pregnancy [19, 20]. This reported difference may be because of the tradition of early marriage and early pregnancy in India. In our study, the literacy rate was high (90%); the possible reason for this could be the site of sample selection, tertiary health care centers. If we consider the primary health center, the literacy level may fall significantly, as mentioned in one of the studies [21]. The majority (60.0%) of the participants were in the second trimester of the pregnancy. The possible reason could be more concern about the fetus in this trimester.

In the present survey, respondents revealed good oral health knowledge and a positive attitude towards oral health; similar results were observed in other studies [22–24]. The possible explanation for this may be that the study respondent was from the high education group. According to the findings of a recent study, most pregnant women (96%) did not receive any information about the impact of oral health on pregnancy from their gynecologist. This result was confirmed by other Australian and European studies [24, 25]. Another crucial finding reported was about dental treatment during pregnancy. The majority of the participants (62.5%) believed that dental treatment should be done before pregnancy, and 32% believed after pregnancy. The possible reason for avoiding treatment during pregnancy could be myths related to dentistry in the Indian population. Indian pregnant women reported that they feared that visiting a dentist during pregnancy was not safe [22]. Another study identified the misconception that deprived oral health is accepted during pregnancy or dental procedures can harm the fetus [26].

When comparing knowledge and practice of oral and dental healthcare, this study revealed that participants were not practicing oral hygiene to a satisfactory level. In the present study, most participants (80.5%) used to brush their teeth once daily. Studies from Europe and Gulf countries reported that 96% to 66% of pregnant women brush their teeth twice daily [26, 27]. The reason behind brushing only once may be more extended family responsibilities and their belief that once a day is sufficient to maintain oral hygiene. The majority of the participants brushed their teeth for one minute or less than one minute. Fluoridated toothpaste was used by most of the respondents, mainly from the higher education group. A similar finding is reported by other studies that identified educated women had better knowledge about fluoride toothpaste and water in preventing tooth decay [21, 28].

About 81% of the participants were willing to visit the dentist only if there was pain and discomfort. The possible cause for this behavior is well explained in an Australian study. The high cost of dental services, lack of awareness among pregnant women about the importance of maternal oral health, safety concerns regarding dental treatment, and time restraints seem to be other restrictive factors [29]. Other studies from countries with free dental care during pregnancy show a fairly low proportion of pregnant women receiving consultation, ranging from 27% in Greece to 33% in the United Kingdom [28]. According to a Malaysian study, pregnant women who knew about the possible consequences of poor oral health reported to the dental office [30].

There are certain limitations in the present study. One limitation is the reliance on self-reported data that is purely subjective, which is often subject to bias. Another limitation is the recruiting method chosen for the study population from the gynecology outpatient department centers. In addition, most participants were from the high education group, which does not reflect the general population where most women are uneducated. Considering all these limitations, we cannot generalize the results obtained to the larger population. Nonetheless, the results would serve as guidelines for designing and specifying proper oral health education for pregnant women.

## CONCLUSION

We observed critical gaps in knowledge and practice of oral and dental healthcare of pregnant women in South India. Pregnant women did not practice oral hygiene as recommended and were reluctant to routine dental visits during pregnancy. Women with lower educational levels knew less about the beneficial effects of fluoride toothpaste. It is very important to understand the knowledge and practice of oral hygiene among pregnant women, given that the maintenance of oral hygiene during pregnancy improves general health. The importance of oral health and its correlation with systemic health, if explained by the gynecologist, will play a crucial role in improving oral hygiene practice and regular dental visits for pregnant women.

## ACKNOWLEDGMENTS

### Conflict of interest

The authors confirm no conflict of interest.

### Ethical approval

The study was approved by the Institutional Review Board of College of Dentistry, King Khalid University, Abha, Saudi Arabia (SRC/ETH/2017-18/057).

### Consent to participate

Written informed consent was obtained from participants before participating in the study.

### Authorship

MAJ, SAS contributed to conceptualizing the study. MA, IK, AYA contributed to the methodology, SAS, MAK, AYA contributed to writing the original draft. MAJ, MA, AYA contributed to editing the manuscript. MAJ, MAK, EA contributed to data collection. SAS, IK, EA contributed to data curation. IK, MAK, EV contributed to data analysis.
